# An Appraisal of Proliferation and Apoptotic Markers in Papillary Thyroid Carcinoma: An Automated Analysis

**DOI:** 10.1371/journal.pone.0148656

**Published:** 2016-02-10

**Authors:** Monika Lamba Saini, Caroline Bouzin, Birgit Weynand, Etienne Marbaix

**Affiliations:** 1 Service d'anatomie pathologique, Cliniques universitaires Saint-Luc, and Institut de Duve, Université catholique de Louvain, Avenue Hippocrate, 10 T-1, B-1200, Bruxelles, Belgium; 2 IREC Imaging Platform (2IP), Institut de Recherche Expérimentale et Clinique, Université catholique de Louvain, Brussels, Belgium; 3 Pathologische Ontleedkunde, Universitair Ziekenhuis Leuven, Herestraat, 49, B-3000, Leuven, Belgium; IPATIMUP/Faculty of Medicine of the University of Porto, PORTUGAL

## Abstract

**Introduction:**

Proliferation and apoptosis are opposing processes by which the cell numbers are kept in a delicate balance, essential for tissue homeostasis, whereas uncontrolled growth of cells is a hallmark of cancer. Papillary thyroid cancer (PTC) is the commonest type of thyroid cancer, with some PTC following an indolent course, whereas the other ones are more aggressive.

**Aim:**

To evaluate respective contribution of proliferation and apoptosis in the tumorigenesis of PTC by automated analysis.

**Materials and Methods:**

We investigated the immunolabeling of phosphorylated histone H3 (pHH3), cyclin D1, active caspase-3, and bcl-2 in thirteen cases each of metastatic PTC, follicular variant of PTC (FVPTC), papillary microcarcinoma (PMC) and well differentiated tumor of uncertain malignant potential (WDT-UMP). FVPTC cases comprised seven encapsulated and six unencapsulated cases.

**Results:**

Proliferation, as assessed by pHH3 and cyclin D1 immunolabeling, was increased in all PTC variants, including the putative precursor lesion WDT-UMP, compared to normal thyroid tissue. pHH3 was immunolabeled in more cells of metastatic PTC than of PMC and of encapsulated FVPTC. Surprisingly, metastatic PTC and unencapsulated FVPTC also demonstrated more cleaved caspase-3 immunolabeled cells than the other types. In contrast, increased expression of bcl-2 protein was seen in normal thyroid areas, encapsulated FVPTC and PMC as compared to metastatic PTC. Metastatic PTC shows higher proliferation than other types of PTC but unexpectedly also higher apoptotic levels. Similar results were also seen with unencapsulated FVPTC, thus suggesting that unencapsulated FVPTC has a potential for adverse outcome. Bcl-2 was immunolabeled in a low percentage of cells in WDT-UMP.

**Conclusions:**

The expression of the proliferative protein pHH3 together with the apoptotic marker cleaved caspase-3 may indicate an aggressive behaviour of PTC and loss of apoptosis inhibition by bcl-2 protein can further amplify the role of these proteins in tumor progression. Both cyclin D1 and bcl-2 could prove to be interesting markers of PTC precursor lesions. Automated/digital image quantification approach helps in refining the diagnostic accuracy.

## Introduction

Papillary thyroid cancer (PTC) is the most common type of thyroid cancer, accounting for 85–90% of all thyroid malignancies [1]. There has been an increasing trend in its incidence, which may be attributed to early diagnosis and a concurrent increase in screening and surveillance intensity.

Uncontrolled growth of cells is a hallmark of cancer and proliferation markers help in deciphering the proliferative potential of the cells. Although proliferation is now widely estimated by the immunohistochemical assessment of the nuclear antigen Ki67, divergent results were found by various groups in thyroid tumors [2–4]. Ki 67 is expressed during all active phases of the cell cycle [1, 5], whereas phosphorylated histone H3 (pHH3) has emerged as a more specific marker of the mitotic phase as the antibody detects the core protein only when phosphorylated at serine 10. Its immunolabeling allows to easily distinguish mitoses from their mimics in hematoxylin and eosin stained histological sections and helps to confidently assess cell proliferation [6, 7]. However, we believe that pHH3 immunolabeling has not yet been used to evaluate cell proliferation in PTC. Cell cycle progression is brought about by cyclin-dependent kinases (CDK) that are activated by cyclins including cyclin D1 and inactivated by CDK inhibitors [8]. Located on chromosome 11q13, cyclin D1 proto-oncogene regulates G1 to S-phase transition in varied cell types from different tissues and has been linked to aggressive behaviour of PTC [9].

Proliferation and apoptosis are opposing processes by which the cell numbers are kept in a delicate balance, essential for tissue homeostasis. Several studies have elucidated a link between apoptosis and cell cycle control [10, 11]. Apoptotic cell death is mediated by caspases, with caspase-3 being their predominant executioner, resulting in DNA fragmentation and nuclear disintegration [12]. Active caspase-3 immunolabeling helps to identify apoptotic cells in tissue sections and is considered more effective than the TUNEL method or morphological assessment [13]. We decided to evaluate apoptosis in PTC and its variants by immunolabeling active caspase-3, which has been previously linked to the stage and aggressive nature of PTC [14]. Bcl-2 is a known inhibitor of apoptosis, and its over-expression may result in proliferation of mutated cells normally scheduled for death [15]. Previous reports have suggested decrease in bcl-2 expression in PTC as compared to normal tissue [16]. Since bcl-2 protein suppresses apoptosis by preventing caspases to carry out the process, it became imperative to assess its immunolabeling pattern in PTC. Study of all these biomarkers is thus likely to increase the understanding of the biological basis of PTC [17].

Recent years have seen rapid advancement in digital pathology with whole slide imaging and automated quantification of the immunostained tissue sections [18]. We have recently demonstrated that automated analysis leads to better validation and reproducibility of immunohistochemical analyses [19]. The American Society of Clinical Oncology and the College of American Pathologists have also approved HER2 image analysis in the evaluation of breast cancer, further emphasizing the importance of digital pathology [20]. We, therefore decided to use this technique to compare the proliferative and apoptotic levels of tumor cells in metastatic PTC of classic type, in follicular variants of PTC (FVPTC), in papillary microcarcinomas (PMC), and in putative PTC precursors, so-called ‘well differentiated tumors of uncertain malignant potential’ (WDT-UMP). To the best of our knowledge, this is the first study that provides an automated assessment of the proliferative capacity and apoptotic potential of cells in these various types of PTC.

## Materials and Methods

### Patients and selection of cases

The study was approved by the Ethics Committee of the Université catholique de Louvain. Cases of PTC and its variants were retrieved from surgical pathology files and 13 cases each of FVPTC, PMC and metastatic PTC were selected along with WDT-UMP, as previously reported [21]. WDT-UMP cases were selected according to criteria described by Williams [22]. The WDT-UMP cases were encapsulated follicular nodules with nuclear features partially characteristic of PTC (i.e., focal nuclear clearing, occasional nuclear grooves, angulated nuclear contours and overlapping nuclei) but the histologic features did not display classic malignant characteristics. FVPTC were selected according to established criteria [23]. Out of the 13 FVPTC cases, seven cases were encapsulated FVPTC and six were unencapsulated FVPTC. Strict criteria were applied for diagnosing encapsulated FVPTC [24]. PMC and infiltrative metastatic PTC were selected according to the criteria of the World Health Organization [25]. All PMC were tumors measuring 1 cm or less in diameter with characteristic PTC type nuclear features. Only the primary tumor was analyzed in metastatic PTC cases.

### Histology and Immunohistochemistry

Formalin-fixed paraffin sections (5μm-thick) were heated at 60°C for 30 min. Deparaffinisation was followed by blocking endogenous peroxidase activity with 3% hydrogen peroxide. A water bath was heated with the staining dish containing 0.01M citrate buffer (pH 5.8) until temperature reaches 100°C. The slides were then put in the staining dish and antigen retrieval was performed by boiling for 75 min. The water bath heating was then turned off and the slides were allowed to cool for 20 min. Sections were then incubated in 0.05% Triton X-100, 0.05M Tris-HCl, pH 7.4 containing 10% goat serum for 30 min to block non-specific binding. This was followed by overnight incubation at room temperature with one of the following primary antibodies: anti-cyclin D1 rabbit monoclonal antibody, diluted 1:25 (RM9104-R7, Thermo Scientific, Fremont, CA, USA); anti-bcl-2 mouse monoclonal antibody, diluted 1:50 (M0887, DakoCytomation); or at 4°C with anti-pHH3 rabbit polyclonal antibody, diluted 1:500 (06–570, Millipore, Billerica, MA, USA), or with anti-active caspase-3 rabbit polyclonal antibody, diluted 1:200 (G748A, Promega, Madison, WI, USA). Sections were washed and then incubated with EnVision+ HRP System^™^ (DakoCytomation) for 60 min. All the immunohistochemical stains were visualized with 3, 3′-diaminobenzidine tetrahydrochloride (DAB) in a hydrogen peroxide solution. Controls were incubated with 0.05M Tris-HCl, pH 7.4 containing 1% goat serum in place of the primary antibody, and no staining was observed.

The apoptotic index was evaluated by counting apoptotic cells on sections stained with hematoxylin and eosin, according to criteria described by Van de Schepop et al [26]. For each case, a map of 100 fields was created at 400-fold magnification and apoptotic bodies were counted in these 100 fields. Results were compared with the immunolabeling of active caspase-3.

### Evaluation of immunostaining

Sections were digitalized at a 20x magnification by SCN400 slide scanner (Leica, Wetzlar, Germany). The tumor tissue in each section was delineated manually and care was taken to exclude tissue folds, bubbles and artefacts from the analysis. Scanned slides were then quantified using Tissue IA (Leica Biosystems, Dublin, Ireland).

Quantification included application of algorithms for nuclear (cyclin D1 and pHH3) or cytoplasmic (bcl-2 and cleaved caspase-3) immunostaining. Color deconvolution was applied to each pixel using hematoxylin and DAB matrices of the software. On the hematoxylin matrice, nuclear parameters (size, heterogeneity, strength of nuclear counterstaining, nuclei density) and cellular parameters (size and cell radius) were adjusted to determine the adequate segmentation. On the DAB matrice, a threshold was adjusted for DAB detection according to intensity (grey values from 0 to 255). These parameters were kept constant throughout the study for each immunostaining.

### Statistical analysis

One-way ANOVA test was performed to test the difference between variants of PTC and with normal thyroid areas for each immunostaining. Mann-Whitney test was then applied to determine between which variants the difference was significant. Difference was considered significant at p<0.05. Correlations were determined by Spearman’s rank correlation coefficient test. Computations were performed and graphs were drawn using GraphPad Prism version 5.04 for Windows (GraphPad Software, La Jolla, CA, USA).

## Results

**Fig 1:**
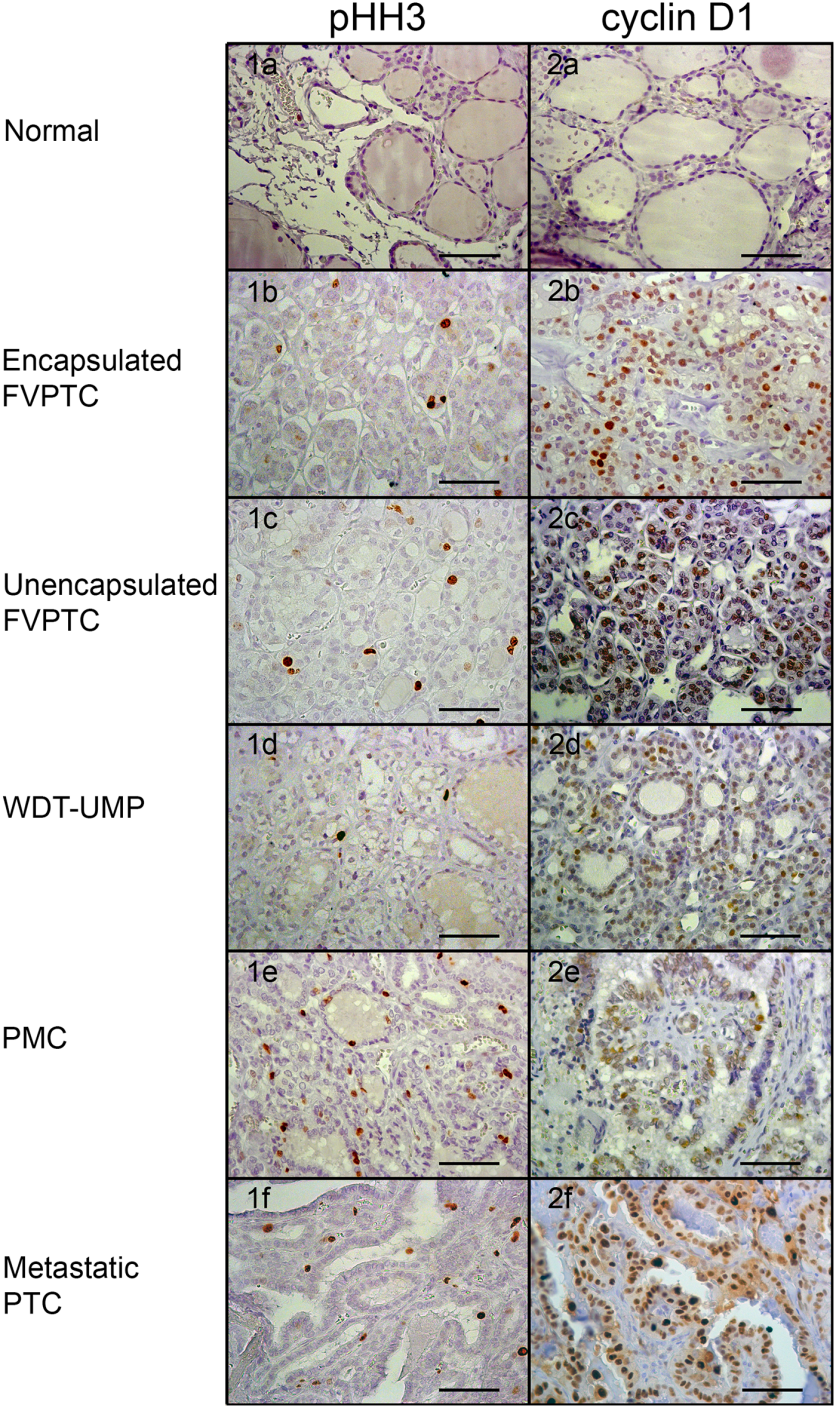
Immunolabeling of proliferative markers. Representative microphotographs of phosphorylated histone H3 (pHH3; 1) and cyclin D1 immunolabeling (2) in normal thyroid tissue (a) and the various types of papillary thyroid carcinomas: encapsulated (b) and unencapsulated (c) follicular variant of papillary thyroid carcinoma (FVPTC), well differentiated tumor of unknown malignant potential (WDT-UMP; d), papillary microcarcinoma (PMC; e) and metastatic papillary thyroid carcinoma (f). Bars correspond to 50 μm.

**Fig 2:**
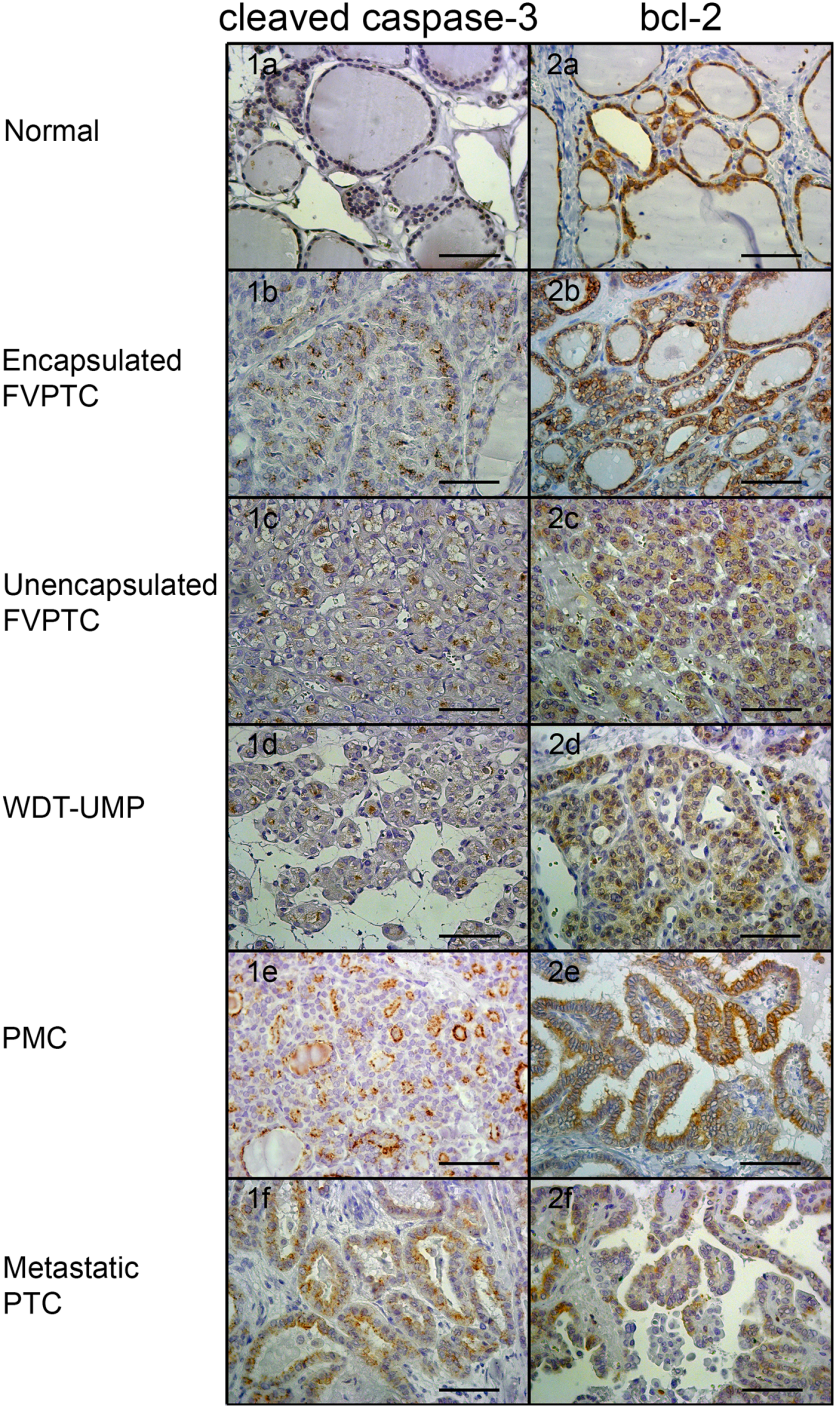
Immunolabeling of apoptosis markers. Representative microphotographs of cleaved caspase-3 (1) and bcl-2 (2) immunolabeling in normal thyroid tissue (a) and the various types of papillary thyroid carcinomas: encapsulated (b) and unencapsulated (c) follicular variant of papillary thyroid carcinoma (FVPTC), well differentiated tumor of unknown malignant potential (WDT-UMP; d), papillary microcarcinoma (PMC; e) and metastatic papillary thyroid carcinoma (f). Bars correspond to 50 μm.

**Fig 3:**
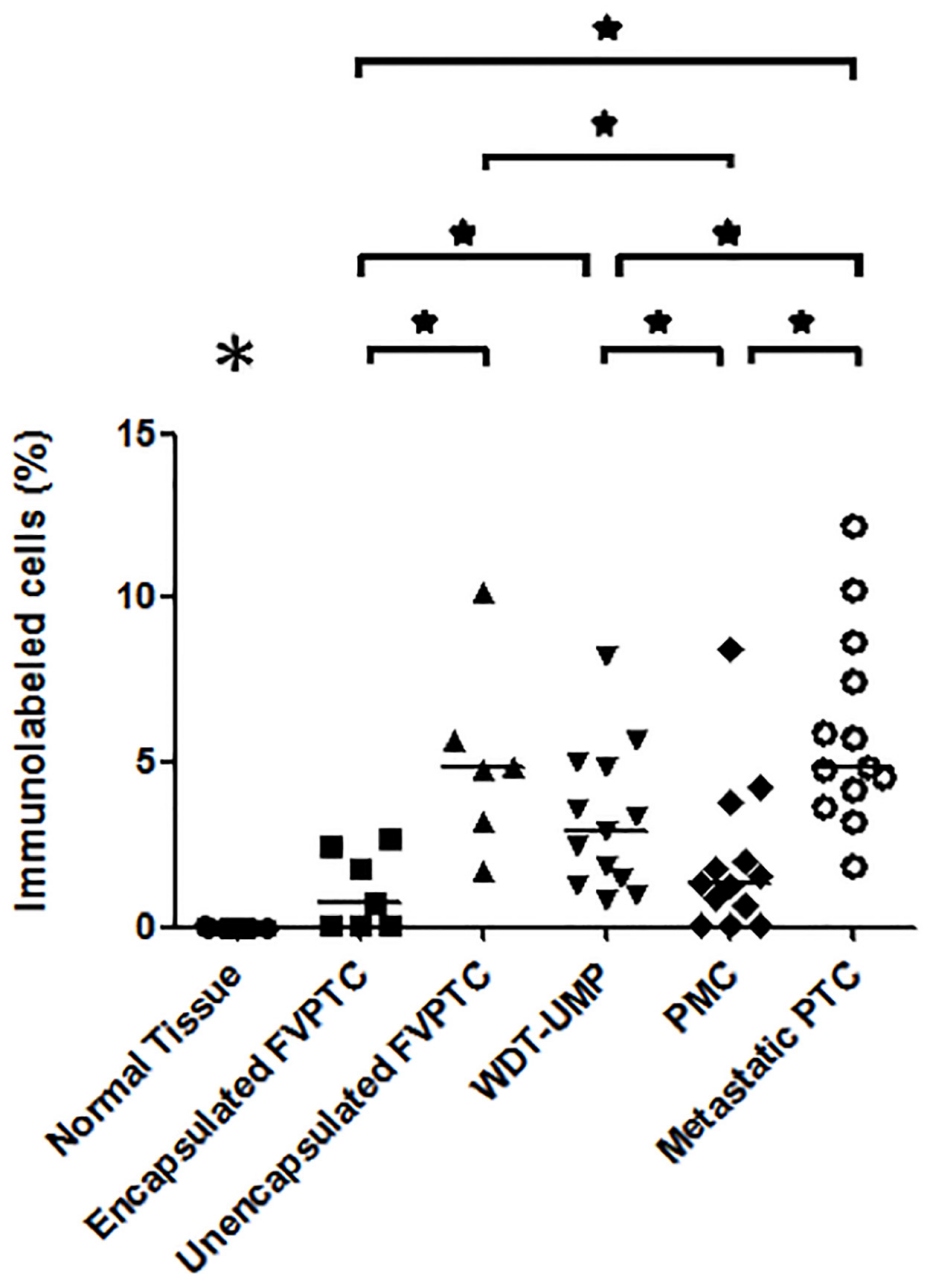
Proportion of tumor cells immunolabeled for phosphorylated histone H3. The percentage of tumor cells showing phosphorylated histone H3 immunolabeling is indicated in normal thyroid tissue and each case of the various types of papillary thyroid carcinomas: encapsulated and unencapsulated follicular variant of papillary thyroid carcinoma (FVPTC), well differentiated tumor of unknown malignant potential (WDT-UMP), papillary microcarcinoma (PMC) and metastatic papillary thyroid carcinoma (metastatic PTC). Bars indicate the medians. *, p<0.05. The large asterisk above normal thyroid tissue indicates significant difference between the normal tissue and any other variant of PTC.

**Fig 4:**
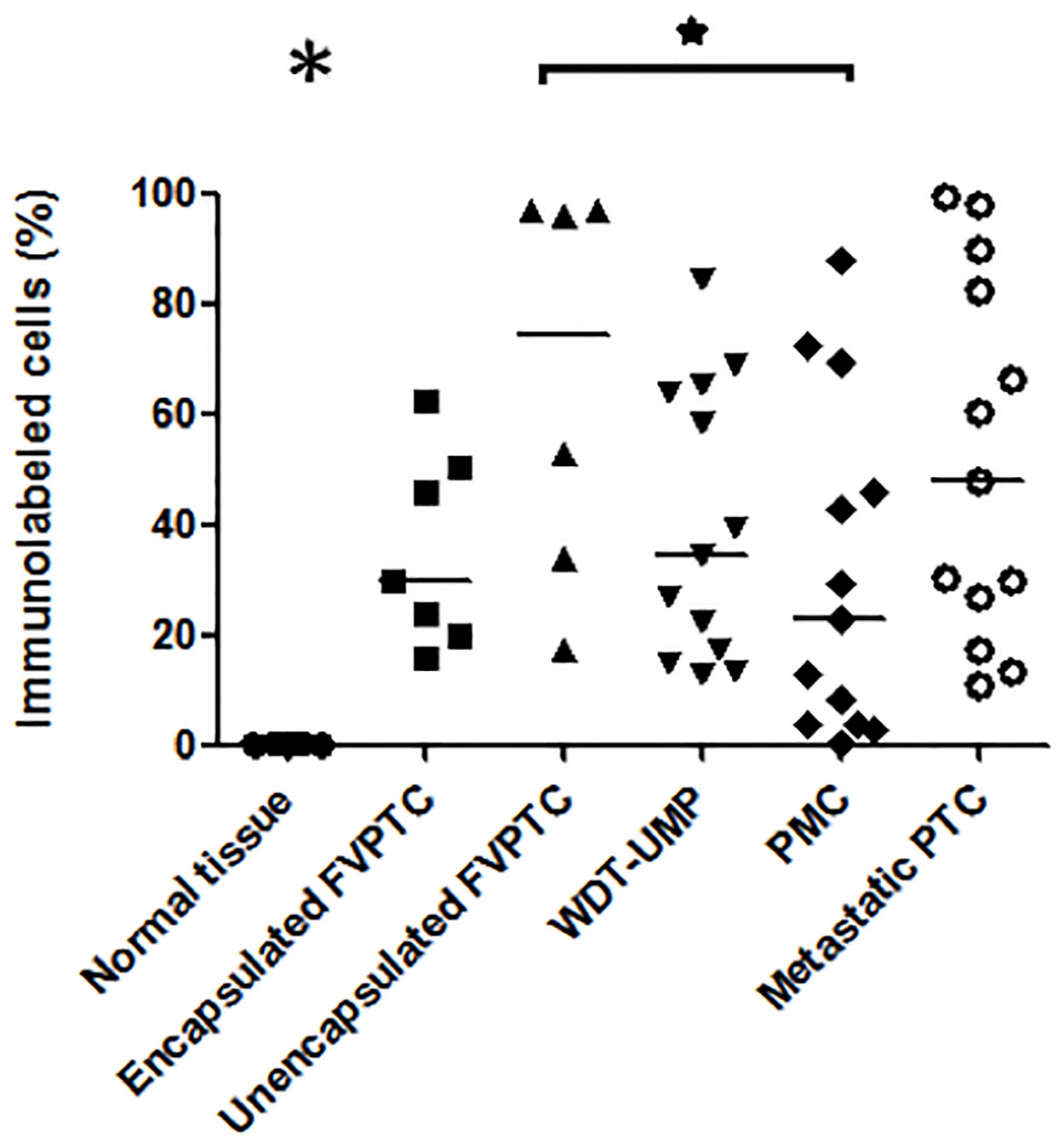
Proportion of tumor cells immunolabeled for cyclin D1. The percentage of tumor cells showing cyclin D1 immunolabeling is indicated in normal thyroid tissue and each case of the various types of papillary thyroid carcinomas: encapsulated and unencapsulated follicular variant of papillary thyroid carcinoma (FVPTC), well differentiated tumor of unknown malignant potential (WDT-UMP), papillary microcarcinoma (PMC) and metastatic papillary thyroid carcinoma (metastatic PTC). Bars indicate the medians. *, p<0.05. The large asterisk above normal thyroid tissue indicates significant difference between the normal tissue and any other variant of PTC.

**Fig 5:**
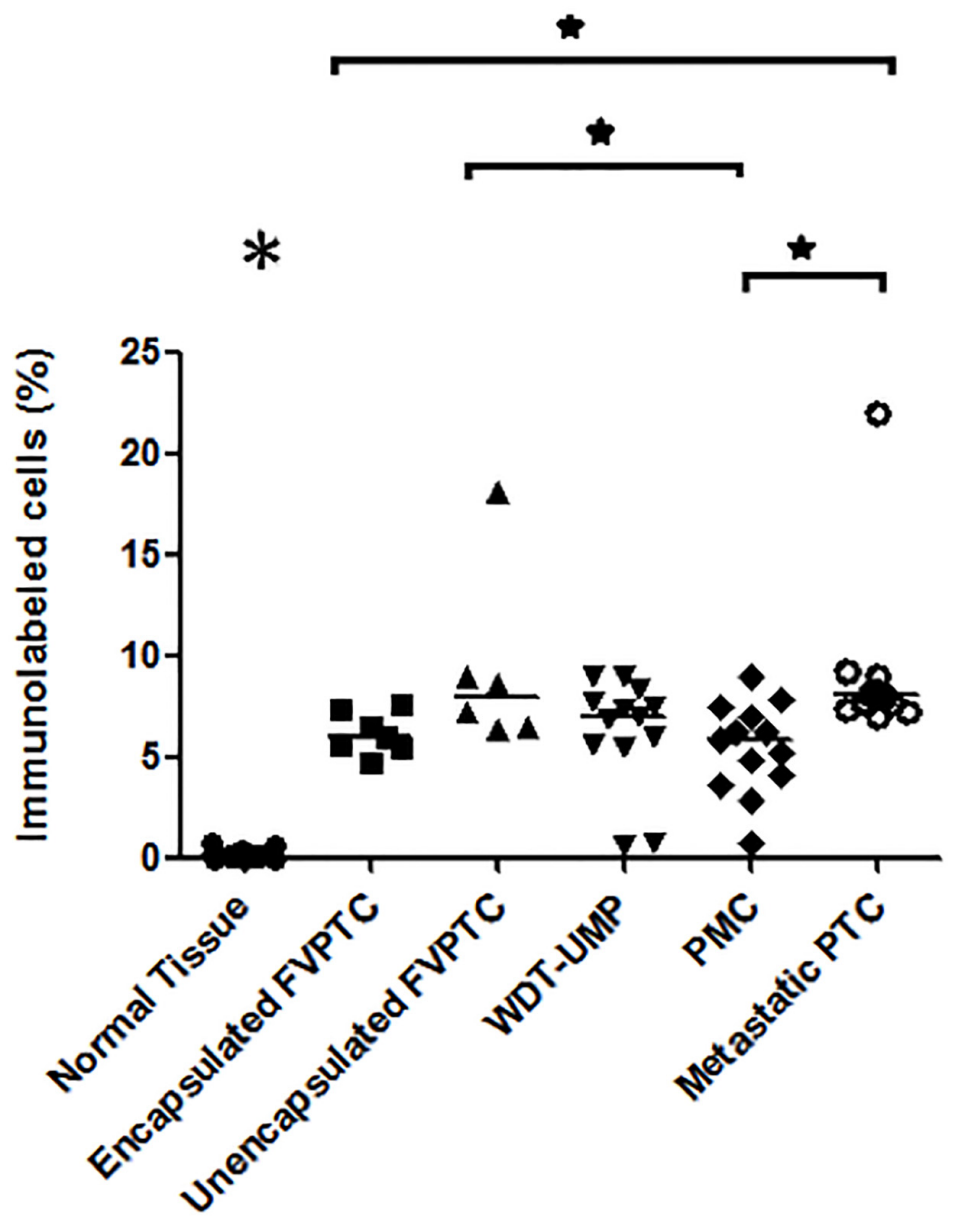
Proportion of tumor cells immunolabeled for cleaved caspase-3. The percentage of tumor cells showing cleaved caspase-3 immunolabeling is indicated in normal thyroid tissue and each case of the various types of papillary thyroid carcinomas: encapsulated and unencapsulated follicular variant of papillary thyroid carcinoma (FVPTC), well differentiated tumor of unknown malignant potential (WDT-UMP), papillary microcarcinoma (PMC) and metastatic papillary thyroid carcinoma (metastatic PTC). Bars indicate the medians. *, p<0.05. The large asterisk above normal thyroid tissue indicates significant difference between the normal tissue and any other variant of PTC.

**Fig 6:**
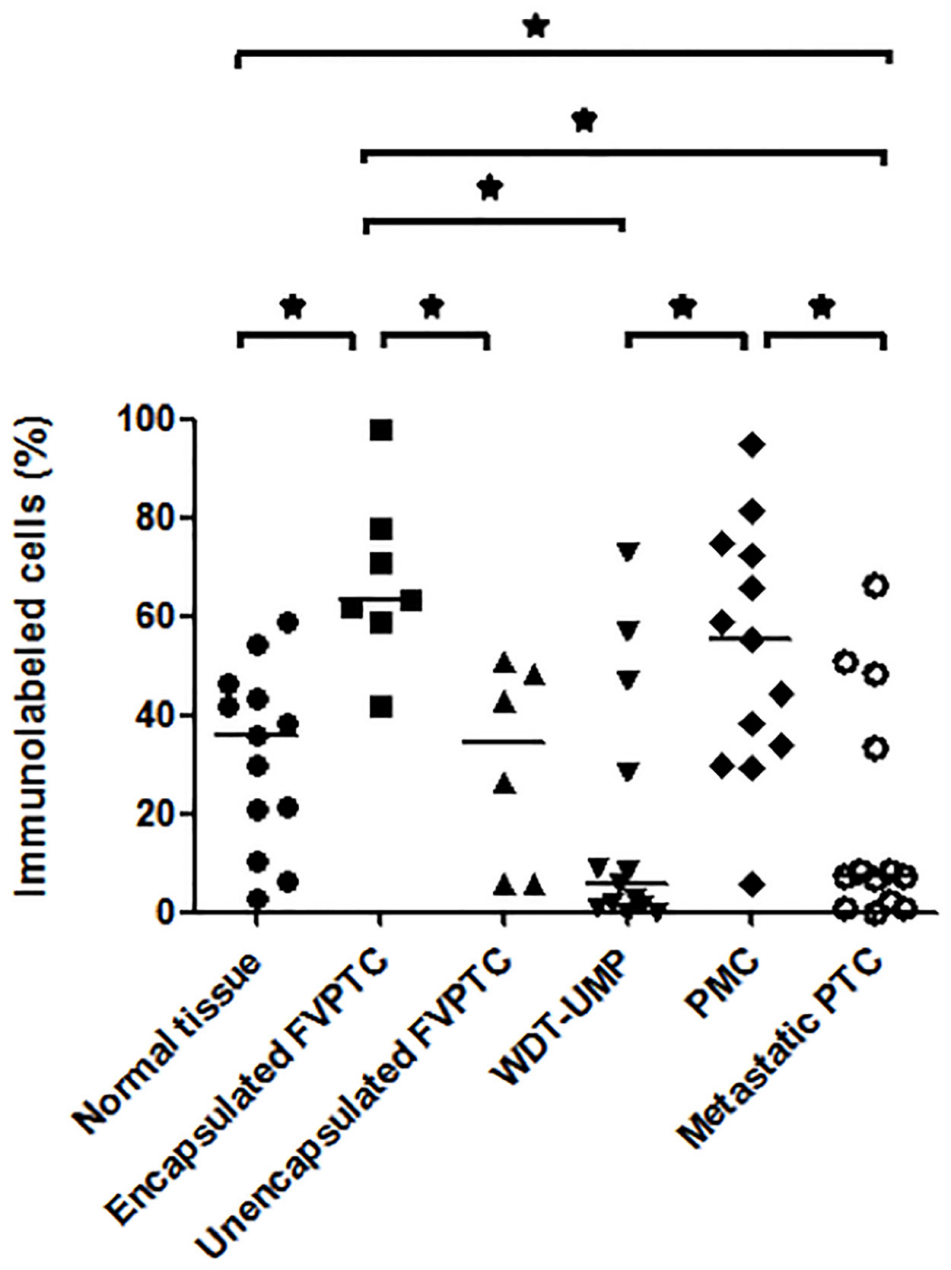
Proportion of tumor cells immunolabeled for bcl-2. The percentage of tumor cells showing bcl-2 immunolabeling is indicated in normal thyroid tissue and each case of the various types of papillary thyroid carcinomas: encapsulated and unencapsulated follicular variant of papillary thyroid carcinoma (FVPTC), well differentiated tumor of unknown malignant potential (WDT-UMP), papillary microcarcinoma (PMC) and metastatic papillary thyroid carcinoma (metastatic PTC). Bars indicate the medians. *, p<0.05.

Proliferative activity was assessed by immunolabeling of pHH3 and cyclin D1 whereas apoptotic potential was evaluated by immunolabeling of cleaved caspase-3, and bcl-2. Representative microphotographs are shown at Figs 1 and 2. Nuclear immunolabeling was seen for pHH3 and cyclin D1 whereas cleaved caspase-3 and bcl-2 were detected in the cytoplasm. Cleaved caspase-3 was seen in the cytoplasm and occasionally in the nucleus of some normal thyroid cells. There was a strong positive correlation between the number of cells immunolabeled for cleaved caspase-3 and the number of apoptotic cells manually counted on hematoxylin and eosin-stained sections (r = 0.76, p<0.0001, S1 Fig). The normal thyroid tissue showed intense cytoplasmic immunolabeling of bcl-2. The average percentage of cells immunolabeled for each marker in normal thyroid tissue and all the variants of PTC is shown in Table 1 whereas Figs 3–6 show the wide distribution of the percentage of immunolabeled cells in each tumor type. Nevertheless, ANOVA test indicated a highly significant difference among all tumor types and normal thyroid tissue for each marker (p<0.0001). Post-hoc non-parametric Mann-Whitney test showed that normal thyroid tissue was significantly different (p<0.05) from every tumor type for each marker, except for bcl-2 where significant difference was found only with encapsulated FVPTC and with metastatic PTC.

**Table 1:** Average immunolabeling levels of proliferative and apoptotic markers (% of total cells).

PTC variants	pHH3	Cyclin D1	Caspase-3	Bcl-2
**Normal thyroid**	0.00	1.50	0.17	32.00
**Encapsulated FVPTC**	1.11	35.47	6.12	67.47
**Unencapsulated FVPTC**	5.06	65.58	9.04	29.27
**WDT-UMP**	3.25	40.13	6.20	17.92
**PMC**	2.01	30.90	5.44	52.59
**Metastatic PTC**	5.96	51.75	9.27	18.50

ANOVA test also showed a highly significant difference among all tumor types for each marker when normal thyroid tissue was excluded from the analysis (p<0.0001). Indeed, metastatic PTC appeared to contain significantly more cells immunolabeled for the proliferative marker pHH3 (Fig 3) than all other PTC variants except unencapsulated FVPTC. The proportion of cells immunolabeled for cyclin D1 (Fig 4) was not different from any other PTC variant but metastatic PTC contained more cells immunolabeled for the apoptotic marker cleaved caspase-3 (Fig 5) and less for the anti-apoptotic bcl-2 (Fig 6) than PMC and encapsulated FVPTC. The unencapsulated FVPTC also contained significantly more cells immunolabeled for the proliferative marker pHH3 than encapsulated FVPTC and PMC (Fig 3) and less cells immunolabeled for the anti-apoptotic bcl-2 than encapsulated FVPTC (Fig 6). In addition, it contained more cells immunolabeled for the proliferative marker cyclin D1 (Fig 4) and the apoptotic marker cleaved caspase-3 than PMC (Fig 5). Unencapsulated FVPTC was not different from metastatic PTC for any marker.

Finally, WDT-UMP appeared to contain more cells immunolabeled for the proliferative marker pHH3 and less cells immunolabeled for the anti-apoptotic marker bcl-2 than PMC and encapsulated FVPTC (Figs 3 and 6). There was no significant difference between PMC and encapsulated FVPTC for any marker.

## Discussion

PTC is the most common endocrine malignancy with an array of architectural/morphological variants. The present work addresses the functionality of proliferative and apoptosis-related biomarkers in PTC variants by an automated morphometric analysis. To the best of our knowledge, this is the only study till date to have made an automated assessment of proliferative and apoptotic markers in these lesions.

Innovations and rapid strides in the field of information technology have given rise to morphometric analyses of histological images, thereby improving our understanding of the biological basis of cancer [27]. Slide digitalization and immunolabeling quantification reduce subjectivity and inter-observer variability, provided quality guidelines are used for pre-analytical steps (delay before tissue fixation, fixation time, section thickness, …), immunostaining technique and automated quantification since algorithms or color matrices may vary from one company/laboratory to the other one [19]. Slide scanner calibration procedures optimize ‘illumination’ and ‘white balance’, which are important parameters affecting image analysis. This leads to high-throughput analysis with constant parameters, continuous data production and better retrieval of results due to better traceability. Comparisons between automated and visual quantification of immunolabeled slides were recently found encouraging [19]. As the accuracy of these technologies improve, the allure and the appeal of digital pathology to be incorporated in routine laboratories increase. The benefits of digital pathology like swift diagnostic opinion for frozen sections, ease of transferring/sharing images, storing and retrieving slides for future use, among others, far outweigh its few negatives. Increased scanning speeds, automated slide loaders and ease of storage have given the required thrust to digital pathology in recent years, prompting the College of American Pathologists and Laboratory Quality Centre to publish guidelines on validating whole slide imaging in diagnostic pathology laboratories [28].

The proliferative potential of the cells has been presented as a valuable tool to assess the prognosis of thyroid tumors [29]. We have previously studied Ki 67 in a similar setting and have found significant immunolabeling in metastatic PTC as compared to normal thyroid areas, WDT-UMP, FVPTC and papillary microcarcinomas [21]. The present automated morphometric study showed an increased proportion of pHH3 immunolabeled cells in metastatic PTC and unencapsulated FVPTC compared to other types of PTC. This suggests a progression in the proliferation rate of neoplastic cells according to evolution of PTC towards metastatic potential. Furthermore, normal thyroid tissue did not show immunolabeling of pHH3, and few cells were immunolabeled in benign adenomatoid nodules compared to all types of PTC (not shown). The mitotic marker pHH3 is linked with chromatin condensation in late G2 and M phases of the cell cycle [30]. We believe pHH3 immunolabeling has not been evaluated earlier in thyroid tumors though it was found to be useful in providing prognostic information in melanocytic lesions [31], bladder cancers [32] and also in invasive breast cancer patients with lymph node metastases [33]. In our hands, pHH3 showed increased expression in tumors with metastatic potential. However, there was no significant difference in the proportion of cells immunolabeled for pHH3 between the unencapsulated FVPTC and the metastatic PTC, thus suggesting that unencapsulated FVPTC has a potential for adverse outcome, as reported by other authors [29, 34]. Cyclin D1 is a positive regulator of the progression from G1 to S phase and its overexpression has been reported in various carcinomas [35] and also linked to aggressive behaviour of thyroid neoplasms [36]. Cyclin D1 was identified as a strong candidate diagnostic marker for PTC and its variants, including PMC and the WDT-UMP lesion, helping to identify it as a putative precursor lesion of PTC [21]. It can also serve as a diagnostic tool in FNAC for early diagnosis of PTC [37]. In the present study, the proportion of cells immunolabeled for cyclin D1 also increased in the different PTC variants as compared to normal thyroid areas, as previously reported [21, 38], but was not significantly different between PTC variants, except between unencapsulated FVPTC and PMC. Thus, proliferative markers like pHH3 but not cyclin D1 show a progressively increased expression from PMC and encapsulated FVPTC to lesions with metastatic potential, lending them a predictive prognostic value, even though PTC has been reported to be an indolent tumor with low proliferation rates [39]. The addition of pHH3 as a biomarker to determine the proliferative capacity in PTCs, along with other markers like Ki67, can help in creation of a proliferation profile, which can be specific and easily reproducible.

Tumorigenesis is dominated by processes that control proliferation and apoptosis of cells [40]. Cleaved caspase-3 leads to proteolysis and ultimately apoptosis of cells. Few studies addressed cleaved caspase-3 in thyroid carcinomas, showing no predictive or prognostic role in thyroid carcinomas [41, 42]. Some studies have also linked the expression of caspase-3 with aggressiveness of the tumor in PTC [14]. Thanks to a sensitive and accurate automatic morphometric analysis, we found a small but significant increase in the proportion of cells showing cleaved caspase-3 immunolabeling in the metastatic PTC compared to encapsulated FVPTC and in both metastatic PTC and unencapsulated FVPTC compared to PMC. Along with pHH3 immunolabeling, this result indicates that aggressive lesions show both high proliferation and high apoptotic potential as well. Similar results have also been observed in other cancers [43, 44]. We also found a strong positive correlation between the apoptotic indices by cleaved caspase-3 immunolabeling and manual counting of apoptotic cells, thereby confirming that cleaved caspase-3 is a good apoptotic marker in PTC [13].

We also explored bcl-2, a well known inhibitor of apoptosis, in the various types of PTC. The normal thyroid tissue showed intense cytoplasmic immunolabeling for bcl-2, and PMC and encapsulated FVPTC demonstrated bcl-2 immunolabeling in more cells as compared to the metastatic lesions, implying that the loss of bcl-2 expression could correlate with increasing aggressive nature and adverse prognosis of thyroid neoplasms. The percentage of cells immunolabeled for bcl-2 was also lower in unencapsulated FVPTC compared to encapsulated FVPTC, as well as surprisingly in WDT-UMP compared to encapsulated FVPTC or PMC, suggesting that a large proportion of our WDT-UMP cases could be precursors of unencapsulated FVPTC. There are few data in literature on the significance of bcl-2 immunolabeling in PTC. Müller-Höcker [45] has reported that absence of bcl-2 staining could be an early event in the formation of oncocytic neoplasms in the thyroid. Expression of bcl-2 as an early oncogenic event in medullary thyroid carcinomas was also reported by Wang et al. [46]. Our study also suggests that bcl-2 could be an invaluable marker to track pathogenic progress of thyroid lesions.

The pro-apoptotic marker cleaved caspase-3 shows an increased immunostaining and the anti-apoptotic marker bcl-2 a decreased expression in lesions with metastatic potential as compared to PMC and encapsulated FVPTC, suggesting that apoptosis related to bcl-2 plays a role in thyroid tumorigenesis. The putative PTC precursor lesion WDT-UMP surprisingly showed higher proportion of cells immunolabeled for pHH3 and lower proportion of cells immunolabeled for bcl-2 than encapsulated FVPTC and PMC, thus prompting further work to understand the reason for this difference. We report high proliferation and high apoptotic rates in metastatic PTC, a fact that has also been demonstrated previously in metastatic prostate cancers [43, 47], breast cancer [48] and pilomatrix carcinoma with lymph node metastases [49]. Wang et al [50] have demonstrated that increased apoptosis is indeed seen in malignant tumors. Altogether, this suggests that the delicate interbalance between proliferation and apoptosis is disrupted leading to tumorigenesis.

In summary, the immunolabeling of the proliferative protein pHH3 together with the apoptotic marker cleaved caspase-3 may indicate an aggressive behaviour of PTC and loss of apoptosis inhibition by bcl-2 protein can further amplify the role of these proteins in tumor progression. Loss of bcl-2 expression in PTC with metastatic potential also indicates a predisposition to unfavourable prognosis. The progress of malignancy and metastatic potential of the cells can be well tracked by assessing the proliferative/apoptotic activity of the PTC cells, as suggested by the present automated morphometric study. Automated/digital image quantification approach helps in refining the diagnostic accuracy.

## Supporting Information

S1 Fig Correlation between cleaved caspase-3 immunolabeling and apoptotic cells. The left panel shows a representative microphotograph of an apoptotic cell (arrow) whereas the right panel shows the correlation between automated analysis of cleaved caspase-3 immunolabeling (percentage) and manual counting of apoptotic cells (absolute values). Spearman’s correlation coefficient was highly significant (p<0.0001).
(TIF)

## References

[1] Davies L, Welch HG. Increasing incidence of thyroid cancer in the United States, 1973–2002. JAMA. 2006;295(18):2164–7. 10.1001/jama.295.18.2164 . 16684987

[2] Saiz AD, Olvera M, Rezk S, Florentine BA, McCourty A, Brynes RK. Immunohistochemical expression of cyclin D1, E2F-1, and Ki-67 in benign and malignant thyroid lesions. J Pathol. 2002;198(2):157–62. 10.1002/path.1185 . 12237874

[3] Ito Y, Miyauchi A, Kakudo K, Hirokawa M, Kobayashi K, Miya A. Prognostic significance of ki-67 labeling index in papillary thyroid carcinoma. World J Surg. 2010;34(12):3015–21. 10.1007/s00268-010-0746-3 . 20703465

[4] Song Q, Wang D, Lou Y, Li C, Fang C, He X, et al Diagnostic significance of CK19, TG, Ki67 and galectin-3 expression for papillary thyroid carcinoma in the northeastern region of China. Diagn Pathol. 2011;6:126 10.1186/1746-1596-6-126 22188859 PMC3264507

[5] Aune G, Stunes AK, Tingulstad S, Salvesen O, Syversen U, Torp SH. The proliferation markers Ki-67/MIB-1, phosphohistone H3, and survivin may contribute in the identification of aggressive ovarian carcinomas. Int J Clin Exp Pathol. 2011;4(5):444–53. 21738816 PMC3127066

[6] Ribalta T, McCutcheon IE, Aldape KD, Bruner JM, Fuller GN. The mitosis-specific antibody anti-phosphohistone-H3 (PHH3) facilitates rapid reliable grading of meningiomas according to WHO 2000 criteria. Am J Surg Pathol. 2004;28(11):1532–6. . 15489659 10.1097/01.pas.0000141389.06925.d5

[7] Tsuta K, Liu DC, Kalhor N, Wistuba II, Moran CA. Using the mitosis-specific marker anti-phosphohistone H3 to assess mitosis in pulmonary neuroendocrine carcinomas. Am J Clin Pathol. 2011;136(2):252–9. 10.1309/AJCPDXFOPXGEF0RP . 21757598

[8] Arora N, Scognamiglio T, Lubitz CC, Moo TA, Kato MA, Zhu B, et al Identification of borderline thyroid tumors by gene expression array analysis. Cancer. 2009;115(23):5421–31. 10.1002/cncr.24616 . 19658182

[9] Fusco A, Chiappetta G, Hui P, Garcia-Rostan G, Golden L, Kinder BK, et al Assessment of RET/PTC oncogene activation and clonality in thyroid nodules with incomplete morphological evidence of papillary carcinoma: a search for the early precursors of papillary cancer. Am J Pathol. 2002;160(6):2157–67. 10.1016/S0002-9440(10)61164-9 12057919 PMC1850819

[10] Fotedar R, Diederich L, Fotedar A. Apoptosis and the cell cycle. Prog Cell Cycle Res. 1996;2:147–63. . 9552392 10.1007/978-1-4615-5873-6_15

[11] Pucci B, Kasten M, Giordano A. Cell cycle and apoptosis. Neoplasia. 2000;2(4):291–9. 11005563 10.1038/sj.neo.7900101 PMC1550296

[12] Slee EA, Adrain C, Martin SJ. Executioner caspase-3, -6, and -7 perform distinct, non-redundant roles during the demolition phase of apoptosis. J Biol Chem. 2001;276(10):7320–6. . 11058599 10.1074/jbc.M008363200

[13] Duan WR, Garner DS, Williams SD, Funckes-Shippy CL, Spath IS, Blomme EA. Comparison of immunohistochemistry for activated caspase-3 and cleaved cytokeratin 18 with the TUNEL method for quantification of apoptosis in histological sections of PC-3 subcutaneous xenografts. J Pathol. 2003;199(2):221–8. 10.1002/path.1289 . 12533835

[14] Mishunina TM, Kalinichenko OV, Tronko MD, Statsenko OA. Caspase-3 activity in papillary thyroid carcinomas. Exp Oncol. 2010;32(4):269–72. . 21270757

[15] Czabotar PE, Lessene G, Strasser A, Adams JM. Control of apoptosis by the BCL-2 protein family: implications for physiology and therapy. Nat Rev Mol Cell Biol. 2014;15(1):49–63. 10.1038/nrm3722 . 24355989

[16] Aksoy M, Giles Y, Kapran Y, Terzioglu T, Tezelman S. Expression of bcl-2 in papillary thyroid cancers and its prognostic value. Acta Chir Belg. 2005;105(6):644–8. . 16438077 10.1080/00015458.2005.11679794

[17] Cvejic D, Selemetjev S, Savin S, Paunovic I, Tatic S. Changes in the balance between proliferation and apoptosis during the progression of malignancy in thyroid tumours. Eur J Histochem. 2009;53(2):65–71. . 19683979 10.4081/ejh.2009.e8 PMC3167284

[18] Ghaznavi F, Evans A, Madabhushi A, Feldman M. Digital imaging in pathology: whole-slide imaging and beyond. Annu Rev Pathol. 2013;8:331–59. 10.1146/annurev-pathol-011811-120902 . 23157334

[19] Bouzin C, Lamba Saini M, Khaing KK, Ambroise J, Marbaix E, Gregoire V, et al Digital pathology: elementary, rapid and reliable automated image analysis. Histopathology. 2015 10.1111/his.12867 . 26386281

[20] Wolff AC, Hammond ME, Hicks DG, Dowsett M, McShane LM, Allison KH, et al Recommendations for human epidermal growth factor receptor 2 testing in breast cancer: American Society of Clinical Oncology/College of American Pathologists clinical practice guideline update. Arch Pathol Lab Med. 2014;138(2):241–56. 10.5858/arpa.2013-0953-SA 24099077 PMC4086638

[21] Lamba Saini M, Weynand B, Rahier J, Mourad M, Hamoir M, Marbaix E. Cyclin D1 in well differentiated thyroid tumour of uncertain malignant potential. Diagn Pathol. 2015;10(1):32 10.1186/s13000-015-0262-8 25907675 PMC4407836

[22] Williams ED. Guest Editorial: Two Proposals Regarding the Terminology of Thyroid Tumors. Int J Surg Pathol. 2000;8(3):181–3. . 11493987 10.1177/106689690000800304

[23] Baloch ZW, LiVolsi VA. Our approach to follicular-patterned lesions of the thyroid. J Clin Pathol. 2007;60(3):244–50. 10.1136/jcp.2006.038604 16798933 PMC1860564

[24] Chan J. Strict criteria should be applied in the diagnosis of encapsulated follicular variant of papillary thyroid carcinoma. Am J Clin Pathol. 2002;117(1):16–8. 10.1309/P7QL-16KQ-QLF4-XW0M . 11791591

[25] DeLellis RAL R. V.; Heitz P. U.; Eng C. World Health Organization Classification of Tumours. Lyon, France: IARC Press; 2004.

[26] van de Schepop HA, de Jong JS, van Diest PJ, Baak JP. Counting of apoptotic cells: a methodological study in invasive breast cancer. Clin Mol Pathol. 1996;49(4):M214–7. 16696077 10.1136/mp.49.4.m214 PMC408061

[27] Kayser G, Kayser K. Quantitative pathology in virtual microscopy: history, applications, perspectives. Acta Histochem. 2013;115(6):527–32. . 23313439 10.1016/j.acthis.2012.12.002

[28] Hamilton PW, Bankhead P, Wang Y, Hutchinson R, Kieran D, McArt DG, et al Digital pathology and image analysis in tissue biomarker research. Methods. 2014;70(1):59–73. . 25034370 10.1016/j.ymeth.2014.06.015

[29] Ghossein R. Problems and controversies in the histopathology of thyroid carcinomas of follicular cell origin. Arch Pathol Lab Med. 2009;133(5):683–91. . 19415942 10.5858/133.5.683

[30] Juan G, Traganos F, James WM, Ray JM, Roberge M, Sauve DM, et al Histone H3 phosphorylation and expression of cyclins A and B1 measured in individual cells during their progression through G2 and mitosis. Cytometry. 1998;32(2):71–7. . 9627219 10.1002/(sici)1097-0320(19980601)32:2<71::aid-cyto1>3.0.co;2-h

[31] Nasr MR, El-Zammar O. Comparison of pHH3, Ki-67, and survivin immunoreactivity in benign and malignant melanocytic lesions. Am J Dermatopathol. 2008;30(2):117–22. . 18360113 10.1097/DAD.0b013e3181624054

[32] Telu KH, Abbaoui B, Thomas-Ahner JM, Zynger DL, Clinton SK, Freitas MA, et al Alterations of histone H1 phosphorylation during bladder carcinogenesis. J Proteome Res. 2013;12(7):3317–26. 10.1021/pr400143x 23675690 PMC3743963

[33] Skaland I, Janssen EA, Gudlaugsson E, Klos J, Kjellevold KH, Soiland H, et al Phosphohistone H3 expression has much stronger prognostic value than classical prognosticators in invasive lymph node-negative breast cancer patients less than 55 years of age. Mod Pathol. 2007;20(12):1307–15. 10.1038/modpathol.3800972 . 17917671

[34] Liu J, Singh B, Tallini G, Carlson DL, Katabi N, Shaha A, et al Follicular variant of papillary thyroid carcinoma: a clinicopathologic study of a problematic entity. Cancer. 2006;107(6):1255–64. 10.1002/cncr.22138 . 16900519

[35] Perisanidis C, Perisanidis B, Wrba F, Brandstetter A, El Gazzar S, Papadogeorgakis N, et al Evaluation of immunohistochemical expression of p53, p21, p27, cyclin D1, and Ki67 in oral and oropharyngeal squamous cell carcinoma. J Oral Pathol Med. 2012;41(1):40–6. 10.1111/j.1600-0714.2011.01071.x . 21883486

[36] Khoo ML, Ezzat S, Freeman JL, Asa SL. Cyclin D1 protein expression predicts metastatic behavior in thyroid papillary microcarcinomas but is not associated with gene amplification. J Clin Endocrinol Metab. 2002;87(4):1810–3. 10.1210/jcem.87.4.8352 . 11932322

[37] Fischer S, Asa SL. Application of immunohistochemistry to thyroid neoplasms. Arch Pathol Lab Med. 2008;132(3):359–72. . 18318579 10.5858/2008-132-359-AOITTN

[38] Wang S, Lloyd RV, Hutzler MJ, Safran MS, Patwardhan NA, Khan A. The role of cell cycle regulatory protein, cyclin D1, in the progression of thyroid cancer. Mod Pathol. 2000;13(8):882–7. 10.1038/modpathol.3880157 . 10955455

[39] Trovisco V, Soares P, Preto A, Castro P, Maximo V, Sobrinho-Simoes M. Molecular genetics of papillary thyroid carcinoma: great expectations. Arq Bras Endocrinol Metabol. 2007;51(5):643–53. . 17891228 10.1590/s0004-27302007000500002

[40] Sinicrope FA, Roddey G, McDonnell TJ, Shen Y, Cleary KR, Stephens LC. Increased apoptosis accompanies neoplastic development in the human colorectum. Clin Cancer Res. 1996;2(12):1999–2006. . 9816159

[41] Zhang HY, Meng X, Du ZX, Fang CQ, Liu GL, Wang HQ, et al Significance of survivin, caspase-3, and VEGF expression in thyroid carcinoma. Clin Exp Med. 2009;9(3):207–13. 10.1007/s10238-009-0031-7 . 19205619

[42] Saffar H, Sanii S, Emami B, Heshmat R, Panah VH, Azimi S, et al Evaluation of MMP2 and Caspase-3 expression in 107 cases of papillary thyroid carcinoma and its association with prognostic factors. Pathol Res Pract. 2013;209(3):195–9. . 23384723 10.1016/j.prp.2012.06.011

[43] Tu H, Jacobs SC, Borkowski A, Kyprianou N. Incidence of apoptosis and cell proliferation in prostate cancer: relationship with TGF-beta1 and bcl-2 expression. Int J Cancer. 1996;69(5):357–63. 10.1002/(SICI)1097-0215(19961021)69:5<357::AID-IJC1>3.0.CO;2-4 . 8900367

[44] Furuya Y, Kawauchi Y, Fuse H. Cell proliferation, apoptosis and prognosis in patients with metastatic prostate cancer. Anticancer Res. 2003;23(1B):577–81. . 12680149

[45] Muller-Hocker J. Immunoreactivity of p53, Ki-67, and Bcl-2 in oncocytic adenomas and carcinomas of the thyroid gland. Hum Pathol. 1999;30(8):926–33. . 10452505 10.1016/s0046-8177(99)90246-0

[46] Wang W, Johansson H, Bergholm U, Wilander E, Grimelius L. Apoptosis and Expression of the Proto-Oncogenes bcl-2 and p53 and the Proliferation Factor Ki-67 in Human Medullary Thyroid Carcinoma. Endocr Pathol. 1996;7(1):37–45. . 12114678 10.1007/BF02739913

[47] Aaltomaa S, Karja V, Lipponen P, Isotalo T, Kankkunen JP, Talja M, et al Expression of Ki-67, cyclin D1 and apoptosis markers correlated with survival in prostate cancer patients treated by radical prostatectomy. Anticancer Res. 2006;26(6C):4873–8. . 17214354

[48] Liu S, Edgerton SM, Moore DH 2nd, Thor AD. Measures of cell turnover (proliferation and apoptosis) and their association with survival in breast cancer. Clin Cancer Res. 2001;7(6):1716–23. . 11410511

[49] Bassarova A, Nesland JM, Sedloev T, Danielsen H, Christova S. Pilomatrix carcinoma with lymph node metastases. J Cutan Pathol. 2004;31(4):330–5. . 15005691 10.1111/j.0303-6987.2004.0178.x

[50] Wang RA, Li QL, Li ZS, Zheng PJ, Zhang HZ, Huang XF, et al Apoptosis drives cancer cells proliferate and metastasize. J Cell Mol Med. 2013;17(1):205–11. 10.1111/j.1582-4934.2012.01663.x 23305095 PMC3823150

