# Supplementary figures and images for: An Appraisal of Proliferation and Apoptotic Markers in Papillary Thyroid Carcinoma: An Automated Analysis
Source: PLoS One. 2016 Feb 10;11(2):e0148656. doi: 10.1371/journal.pone.0148656 (PMC4749175; doi:10.1371/journal.pone.0148656)

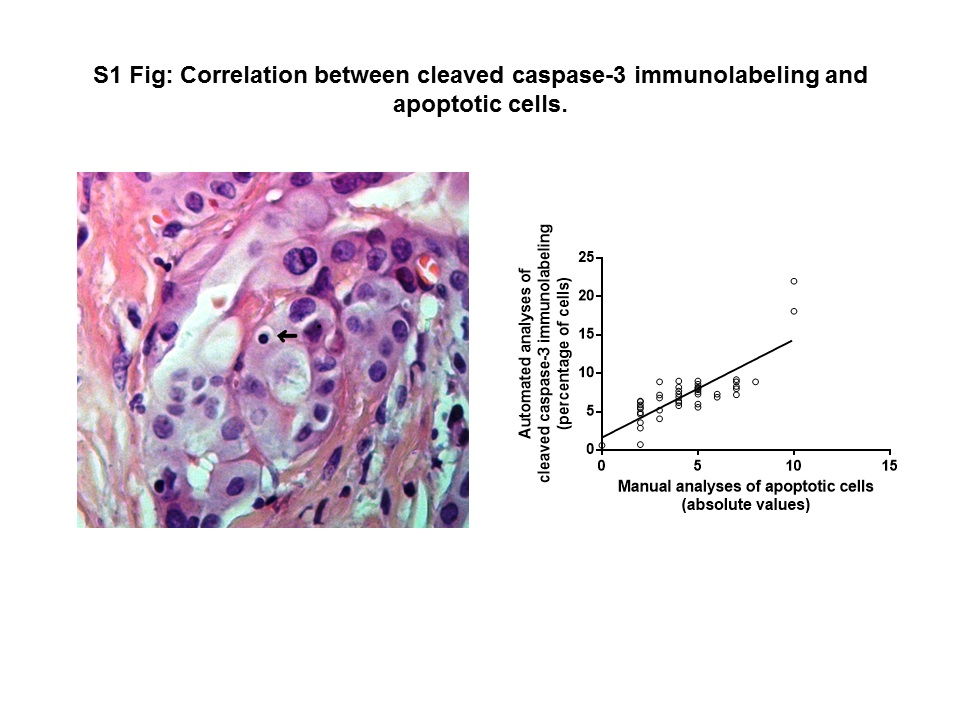

Supplement: S1 Fig — The left panel shows a representative microphotograph of an apoptotic cell (arrow) whereas the right panel shows the correlation between automated analysis of cleaved caspase-3 immunolabeling (percentage) and manual counting of apoptotic cells (absolute values). Spearman’s correlation coefficient was highly significant (p<0.0001). (TIF) [file pone.0148656.s001.tif]
